# A Cluster-Based Architecture to Structure the Topology of Parallel Wireless Sensor Networks

**DOI:** 10.3390/s91210513

**Published:** 2009-12-23

**Authors:** Jaime Lloret, Miguel Garcia, Diana Bri, Juan R. Diaz

**Affiliations:** Departamento de Comunicaciones, Universidad Politécnica de Valencia. Camino Vera s/n, 46022, Valencia, Spain; E-Mails: migarpi@posgrado.upv.es (M.G.); diabrmo@posgrado.upv.es (D.B.); juadasan@dcom.upv.es (J.R.D.)

**Keywords:** sensor algorithms, sensor network, clusters architecture

## Abstract

A wireless sensor network is a self-configuring network of mobile nodes connected by wireless links where the nodes have limited capacity and energy. In many cases, the application environment requires the design of an exclusive network topology for a particular case. Cluster-based network developments and proposals in existence have been designed to build a network for just one type of node, where all nodes can communicate with any other nodes in their coverage area. Let us suppose a set of clusters of sensor nodes where each cluster is formed by different types of nodes (e.g., they could be classified by the sensed parameter using different transmitting interfaces, by the node profile or by the type of device: laptops, PDAs, sensor *etc.*) and exclusive networks, as virtual networks, are needed with the same type of sensed data, or the same type of devices, or even the same type of profiles. In this paper, we propose an algorithm that is able to structure the topology of different wireless sensor networks to coexist in the same environment. It allows control and management of the topology of each network. The architecture operation and the protocol messages will be described. Measurements from a real test-bench will show that the designed protocol has low bandwidth consumption and also demonstrates the viability and the scalability of the proposed architecture. Our ccluster-based algorithm is compared with other algorithms reported in the literature in terms of architecture and protocol measurements.

## 1. Introduction and Related Works

Wireless sensor networks (WSNs) are a large number of small devices capable of executing sensing, data processing and communication tasks. As sensor nodes may be placed everywhere, this type of network can be applied to multiple scenarios [1]. e.g., in healthcare [2], where they are used to monitor and assist disabled patients, habitat monitoring [3], disaster management [4], and even for commercial applications such as managing an inventory, monitoring product quality, surveillance, and target tracking [5].

In cluster based architectures, mobile nodes are divided into virtual groups. Each cluster has adjacencies with other clusters. All the clusters have the same rules. A cluster can be made up of a Cluster Head node, Cluster Gateways and Cluster Members [6]. The Cluster Head node is the parent node of the cluster, which manages and checks the status of the links in the cluster, and routes the information to the right clusters. Inter cluster data transfer takes place through the cluster gateways [7]. Cluster members are the rest of the nodes in a cluster. In this kind of network, Cluster Head nodes are used to control the cluster and the size of the cluster is usually about one or two hops from the Cluster Head node. A cluster member does not have inter-cluster links, only cluster gateways.

There are many cluster based architectures [8]. Sensor networks clustering schemes can be classified according to several criteria. For example, they can be classified according to whether the architectures are based on Cluster Head [9] or on Non Cluster Head [10]. The first architecture needs a Cluster Head to control and manage the group, and the second one does not have a specific node to perform this task. Another way to differentiate the cluster-based architectures is observing the hop distance between node pairs in a cluster. The schedules can be divided into 1-hop clustering [11], multi-hop clustering [12] or multilevel clustering [13]. The maintenance of the hierarchical multilevel requires heavy communication overheads due to random change of multilevel topology. By contrast, the cluster head of single level clustering is simple, since it only tracks local topology changes due to host mobility.

In addition to these classification criteria, reference [8] presents another classification based on the objectives of the clustering protocols. There are six clustering schemes: dominating-set-based (DS-based) clustering [14], low-maintenance clustering [9], mobility-aware clustering [15], energy-efficient clustering [10,16], load-balancing clustering [17] and combined-metrics based clustering [11].

The clustering architectures provide many benefits. Reference [18] shows the most important features of cluster-based architectures over *ad hoc* and sensor networks. The last feature is strongly linked with energy conservation, given that clustered wireless sensor networks offer two major advantages over their non-clustered counterparts; firstly, clustered wireless sensor networks are capable of reducing the volume of inter-node communication by localizing data transmission within the formed clusters and decreasing the number of transmissions to the sink node; secondly, clustered wireless sensor networks are capable of extending the nodes' sleep times by allowing cluster heads to coordinate and optimize the activities.

In the literature several cluster deployments and proposals can be found. One of them is the one presented in [19], which proposes a cluster-based system to overcome problems of bottleneck and poor scalability. In [20], Zou and Chakrabarty proposed a cluster-based distributed sensor network deployment and target localization to enhance the coverage after an initial random placement of sensors.

All these works use a routing protocol inside the cluster [21,22] or they use the cluster to build a unique protocol for the entire network (such as the one presented by Lee *et al.* in [23], where the cluster is used for authentication), but none of the protocols seen use cluster-based schemes to build different networks. So, this is the first time that different sensor networks have been built using cluster-based schemes.

This paper presents a proposal where nodes from different clusters have to communicate in order to build different wireless sensor networks (such as virtual networks). In order to control and manage the system, some limitation parameters have been added. Connections will be established if they are close enough and only if they are the same type of sensor. The main contribution of our work is the design and verification of a new protocol and its comparison with other protocols in existence. There is no proposal in the literature with a similar purpose to the one presented in this paper.

This paper is structured as follows. Section 2 formulates the problem, explains which issue has to be solved and presents some application environments where our proposal can be used. Section 3 describes our proposal. Its scalability is demonstrated in Section 4. Section 5 explains how it operates, how it provides fault tolerance and shows the protocol messages. Section 6 gives the measurements obtained from a test bench and shows the fault tolerant procedure. The comparison of the proposed protocol with other existing cluster-based protocols is shown in Section 7. Finally, Section 8 gives the conclusions and future works.

## 2. Problem Formulation and Application Environments

Let us suppose an environment where a great variety of sensors must be scattered to take measurements from the environment or the same type of sensor but with different types of profiles. Let us suppose that the whole area is divided into zones and each zone has one or several types of sensors (humidity, temperature, wind, movement, *etc.*) organized by a central node as a cluster. Let us also suppose that exclusive networks with the same type of sensors (network of temperature sensors, network of wind sensors, network of humidity sensors, *etc.*) are needed. An example could be the use of clusters of different sensors for each tree in a forest. So, there will be as many networks as types of sensors. It could be also used to create virtual wireless sensor networks (the same concept as Virtual Local Area Networks in wired networks).

Some examples given to explain the form of that exclusive network between nodes from different clusters are the following:

- They could have a different transceiver to connect to other cluster nodes but the same transceiver to connect to the cluster head node.
- They could use a different wireless protocol to connect to other cluster nodes but the same transceiver to connect to the cluster head node.
- They could be using different types of technology to connect to other cluster nodes but the same transceiver to connect to the cluster head node.
- They could be transmitting different types of data that is not understandable by other types of nodes, only by the same type of node and the cluster head node.
- They could have different types of profiles.
- They could be different types of devices.

Moreover, there are some statements that must be added:

- When a new sensor joins the architecture, it will belong to the zone of its nearest central cluster sensor.
- Due to processing consumption issues, the number of connections to the central cluster sensor should be limited, so when it reaches the maximum number of connections, the new sensor has to create a new cluster.
- Sensors will have connections only with the same type of sensors of neighboring groups in a predefined distance or coverage area, but not with nodes from other groups that are not neighbors.
- For energy saving purposes, when there are several sensors from other clusters in the sensor's coverage area, the one with higher capacity (which depends on the energy between other parameters that will be presented later) will be chosen as a neighbor.
- The network formed by sensors of the same type will have its own routing protocol algorithm.

Taking into account the aforementioned premises, several application environments can be found. Some of them are the following:

- It could be used in any kind of system where an event or alarm is based on what is happening in a specific zone, but conditioned to the events that are happening in neighboring zones. One example is a group-based system to measure the environmental impact of a place (forest, marine reef, *etc.*). It could be better measured if the measurements are taken from the plants and from the trees in that place with different type of sensors. Each kind of measurement could be taken from different groups of sensors, but those groups of sensors have to be connected with the same type of sensors in order to estimate the whole environmental impact.
- It could be used in body area sensor networks. The devices used to sense the body could be several types of sensor (pulse sensors, skin sensors, sweat sensors, *etc.*). A sensor may need to be connected with the same type of sensors of other zones of the body to form their specialized network in order to check the measurements of a specific parameter.
- It could be used to build networks of sensors with the same profile that come from different communities (each community will be a cluster).
- It could be used to build networks whose cluster can be formed by different types of devices such as mobiles, PDAs, PCs, sensors, but the requirement is for networks formed by the same type of devices.
- It could be used for virtual wireless sensor network creation too.

## 3. Architecture Proposal Description

From the logical point of view, our proposal is based on a two-layer model involving an organization layer and a distribution layer. All clusters must have both layers. Sensor devices (henceforth referred to as “nodes”) in the organization layer are called Cluster Heads (CH). Although they have sensing capacities, they are the ones with higher capabilities of the cluster (how they are elected is defined later). The distribution layer is formed by cluster member nodes and cluster gateway nodes, henceforth called CMs. CHs also have CM capabilities. The same physical and MAC layers are used between CHs and between CMs and their CHs. CMs could use the same or have another type of physical and MAC layers, using different wireless transceivers, for their exclusive network [24].

CHs organize and control the CMs in their cluster and all CMs have to establish a connection with a CH to join its cluster. This connection can be established only if the distance between them is shorter than or equal to a predefined value. In the rest of the paper, the distance will be considered as one of the limiting parameters to establish connections, but it can be changed by the Received Signal Strength Indication (RSSI) value or by the Signal Noise Ratio (SNR) value or by any other parameter that could be used to know if the candidate neighbor is reachable. In our design only one CH per cluster has been provided, but more could be added for scalability purposes, using the algorithm presented by the same authors of this paper in reference [25].

CHs have connections with some CHs of others clusters. OLSR [26] has been chosen as the routing protocol to route information between CHs, but it could be changed for any other routing algorithm such as AODV [27], DSR [28] or TORA [29]. The organization layer is used to organize connections between CMs of different clusters. There are several types of CMs in a cluster, depending on what they are sensing, the profile, or the type of device. CMs have connections with the same type CMs of other clusters only if the distance between them is shorter than or equal to a predefined value. CHs can be any type of *ad hoc* device or sensor. They can communicate with CMs of other clusters because they are CMs but only if they are the same type of CMs.

The number of clusters in the network is determined by the extension that is to be covered by the whole network. If a new zone needs to be covered, a new cluster has to be added. Although many types of sensors or types of devices can be added to any cluster, the application of the 20/80 rule (20% of CHs, 80% of CMs) is suggested [30].

**Figure 1.**
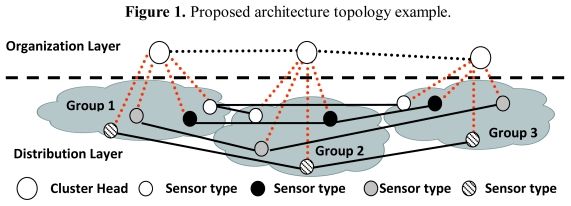
Proposed architecture topology example.

An example of the architecture proposed is shown in Figure 1. Although a CH is in both layers, they have been placed in the organization layer just to clarify the Figure. CHs have connections with some CHs from other clusters (lines formed by black points). All CMs have a connection with the CH of its cluster (lines formed by red points) and with the selected CMs from other clusters (solid black lines).

### 3.1. Identifiers and Predefined Parameters

Every cluster has an identifier called *clusterID*. When a new node joins a cluster it acquires a unique 32-bit node identifier called *nodeID* from the CH. The first node in a cluster will be the CH and will have *nodeID* = 0x01. Once the CH of the new cluster has contacted with other CHs in the network, it will acquire the first available *clusterID* and, then, it will try to connect with the same type of CMs from other clusters. All nodes in a cluster have the same *clusterID*. Any new node will join the cluster whose CH is closest.

Every new node must have the following parameters to join the proposed architecture:

- Max_con: Maximum number of supported connections from other nodes of the distribution layer.
- Type: It identifies the type of node.
- Max_distance: It is the maximum distance to be a neighbor. It is always shorter than or equal to the coverage area radius. It can be changed by the Received Signal Strength Indication (RSSI) value or by the Signal Noise Ratio (SNR) value. It is applied only to establish connections between CHs and their CMs and between CMs, but not between CHs because CH must have as many connections with other CHs as possible. We have proved in other works [31] that although inductive and hybrid methods provide higher reliable values, we are considering a hard environment where there might not be any previous training phase.
- Position: It could be given manually or by GPS.
- It will have other parameters that vary along its existence in the architecture:
- Available_con: Number of available connections with other nodes of the distribution layer.
- E: % of energy consumption.
- L: % of available load. The load is the quantity of tasks the node is able to carry out at one time.

Two parameters have been defined to be used for the operation of the architecture.

### 3.2. δ Parameter

**Figure 2.**
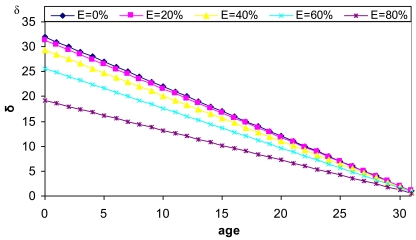
*δ* values as a function of node age.

It depends on the node available energy and its age in the system (the lower *nodeID*, the older the node is). It is used to ascertain which node is the best one to be a Cluster Head node. This would seem to be anomalous since the oldest node should be the lowest energy node, but this parameter appears to consolidate the most stable nodes as the CHs (new ones could be mobile nodes or even with lower energy). So, when those in the cluster have low energy, only new nodes with very high available energy will be preferred. A node with higher available energy and older will have higher δ. Equation 1 defines the *δ* parameter:

(1) $$\delta =(32-\text{age})\sqrt{1-\frac{E^{2}}{K_{1}}}$$

where *age* = log2(*nodeID*), so age varies from 0 to 31. *E* values vary from 0% to 100%. *E* = 0 indicates it is fully charged and *E* = 100 indicates it is fully discharged. *K*_1_ defines the minimum value of energy remaining in a node to be suitable for being selected as a neighbour. Figure 2 shows *δ* parameter values as a function of the node age for different available energy values. We have fixed *K*_1_ = 104 to have *δ* within the desired range values.

### 3.3. λ Parameter

**Figure 3.**
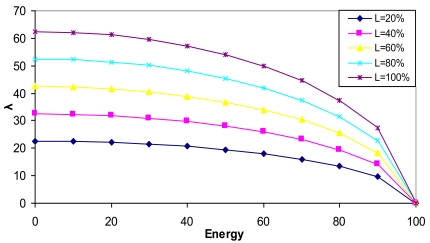
*λ* values as a function of node energy.

It is the capacity of a node. It is used by the CMs to determine the best CM to connect with when there are several choices. *λ* parameter depends on the node's number of available connections (*Available_Con*), its maximum number of connections (*Max_Con*), its % of available load (*L*) and its % of available energy (*E*). It is defined by Equation 2:

(2) $$\lambda =\frac{\text{Available}\_\text{Con}\cdot L+K_{2}}{\text{Max}\_\text{Con}}\cdot \sqrt{1-\frac{E^{2}}{K_{1}}}$$

where 0 ≤ *Available_Con* ≤ *Max_Con. L* is the available load and *E* is the energy consumption. *L* and *E* values vary from 0 to 100, according to the state of the node. An energy consumption of 0 indicates it is fully charged and a value of 100 indicates that it is fully discharged. *K*_1_ is defined as it was for *δ* parameter and *K*_2_ gives *λ* values different from 0 in case of *L* = 0 or *Available_Con* = 0. The root is excluded from the division because when the node is fully discharged, *λ* parameter has to be 0. We have considered *K*_2_ = 100 to get *λ* into desired values. Figure 3 shows *λ* parameter values when the maximum number of links for a node is 8 and all have the same available number of links (*Available_Con* = 4) as a function of the node available energy for different load values. It shows that as the Energy is being consumed, *λ* parameter is decreasing, but when it receives 80% of consumption, it decreases drastically, so the node is more likely to be chosen as a neighbor, in case of more available energy. Figure 3 also shows that a node with higher bandwidth is preferred.

## 4. Scalability

It is known that cluster based systems are more scalable than other systems. This section shows why our proposal scales better than other proposals. First, we have to take into account that computation is much cheaper than communication in terms of energy dissipation [32]. So, what is desired is architecture with fewer retransmissions. This will imply a saving in energy of the whole system and it will give more scalability to the architecture.

Let a network of nodes *G* = (*V, E*) be, where V is the set of nodes and *E* is the set of connections between nodes. Let *k* be a finite number of disjoint clusters of *V*, so *V* = (*V_k_*) and there is no node in two or more subsets (∩*V_k_* = 0), *i.e.*, there are not overlapping nodes. Let us suppose *N* = |*V*| (the number of nodes of *V*) uniformly distributed in a region. Let us suppose that there is just one cluster head node per cluster, so there are k head clusters in the whole network. Equation 3 gives the number of nodes:

(3) $$N=\sum_{i=1}^{k}|V_{k}|\;$$

and the average number of neighbors of a cluster head will be given by Equation 4:

(4) $$\text{Average}=\frac{N}{k}-1$$

**Figure 4.**
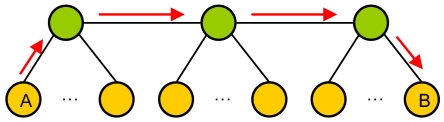
1-level cluster.

**Figure 5.**
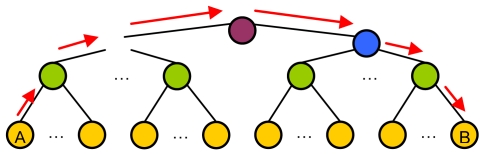
P-level cluster.

**Figure 6.**
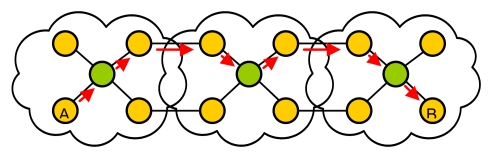
Planar cluster.

**Figure 7.**
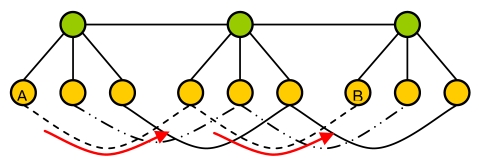
Our proposal (in the worst case).

Four main types of cluster architectures can be distinguished:

- 1-level cluster (see Figure 4).
- P-level cluster (see Figure 5).
- Planar cluster with 1 hop (see Figure 6).
- Our proposal (see Figure 7). We have considered the worst case where the communication has to be done through a node of an intermediate cluster (it could be done directly if it is reachable).

The diameter of a network (d) is defined as the length of the delay-optimal path between the two farthest nodes. Figures 4–7 also show the path between the two farthest nodes.

Table 1 shows the diameter for each type of cluster.

**Figure 8.**
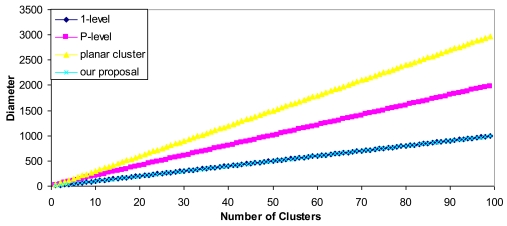
Diameter.

Figure 8 shows the diameter of each cluster architecture (we have considered *K* = *P* for the P-level cluster).

Figure 8 demonstrates fewer hops are needed in our architecture than for the others, so the total routing overhead of the network is reduced and fewer retransmissions are needed, thus more energy is saved. This implies that our proposal scales better than the others.

## 5. Architecture Proposal Operation and Fault Tolerance

**Figure 9.**
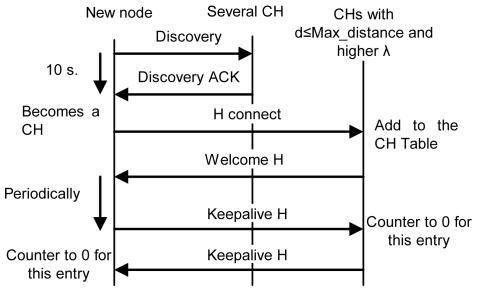
Protocol operation for a new CH.

**Figure 10.**
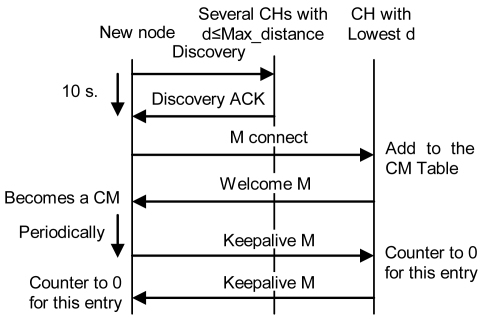
Protocol op0eration for a new CM.

In order to join the architecture, the new node broadcasts a “Discovery” message. CHs will reply with a “Discovery ACK” message with their position and *λ* parameter. There could be three possibilities:

1. If it does not receive any reply within 10 seconds, it becomes a CH, so it creates the cluster and waits for new nodes. Ten seconds have been chosen because it is enough time to receive a reply from a near node. Later replies will be from nodes which are either too far or too busy.
2. If it receives some replies, but none of them are at a distance lower than the Max_distance, it becomes a CH and sends an “H connect” message to establish connections with selected CHs (based on their *λ* parameter). If the other CH confirms that connection, it adds this entry to its CH table and sends a “Welcome H” message with the last *clusterID* in the network. The CH will choose the next available *clusterID* for its cluster. Then, the new CH will send “Keepalive H” messages periodically with its *clusterID* to its neighbor CHs to indicate it is alive. If a CH does not receive a “Keepalive H” message from a CH for a dead time, it would erase that entry from the database. “Keepalive H” messages contain sender's *clusterID* and *λ* parameter. The CH also follows the new CM process described later. Messages sent in this case, when there is a new CH, are shown in Figure 9. Once the discovering process has finished CH node's network works as a regular OLSR network.
3. If it receives one or several replies and all of them are within a distance lower than the Max_distance, it chooses the closest CH and in case of a draw, the one with highest *λ* parameter. Then, it sends an “M connect” message to establish a connection with the selected CH. If the other CH confirms that connection because it has not reached the maximum number of connections, it adds this entry to its CM table, sends a “Welcome M” message and the new node becomes a CM. If the CH does not agree the connection, the new node sends an “M connect” message to the second best CH and follows the same steps. This process is repeated until the new node reaches the last option. If the last option does not confirm the connection, the new node becomes a CH and follows the steps explained in case 2. When a CM receives a “Welcome M” message, it will know which its cluster is. It will send “Keepalive M” messages periodically to the CH to indicate it is alive. If the CH does not receive a “Keepalive M” message from a CM for a dead time, it will erase that entry from the database. “Keepalive M” messages contain the *nodeID* of the sender, its λ and its δ parameters. Steps followed when there is a new CM in this third case are shown in Figure 10.

**Figure 11.**
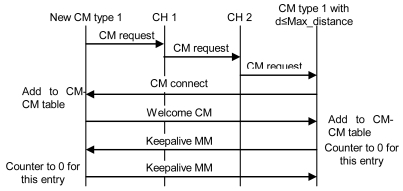
Protocol operation to establish a connection with a CM of another cluster.

**Figure 12.**
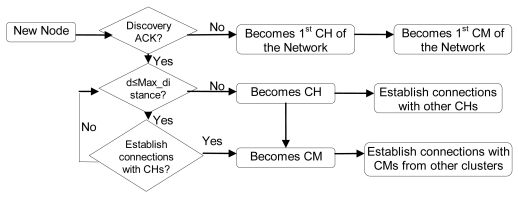
Flowchart of the architecture operation.

When there is a new CM in a cluster, it has to establish connections with CMs from other clusters. They must be the same type of CM, and then, the distance between them has to be lower than or equal to than Max_distance (remember that it could be changed by the Received Signal Strength Indication (RSSI) value or by the Signal Noise Ratio (SNR) value or by any other parameter that can be used to know if the neighbor is reachable). First, it has to send a “CM request” message to the CH of its cluster. This message has the requester's CM type and its position. When the CH receives this message, it changes the *nodeID* by the *clusterID* in the message and forwards it. It is sent only to neighboring CHs because they are the only ones that will meet the Max_distance requisites. When a CH of another cluster receives that message, it sends the message to the appropriate CM (based on the type of CM). CMs that have not reached the Max_con value will send a “CM connect” message to the new CM. If it receives more than one “CM connect” messages from the same cluster, first it will choose the closest one, and in case of a draw, the one with highest *λ* parameter. Then, it will add these neighbors in its CM-CM table and will send them a “Welcome CM” message. They will add this entry to its CM-CM table. Finally, both will send “keepalive MM” messages periodically to indicate that they are still alive. If any one of them does not receive a “keepalive MM” message for a dead time, it will erase this entry from its database, so it will send a new “CM request” for this cluster. “Keepalive MM” contain the clusterID of the sender and its *λ* parameter. If the CM does not find any CM of the same type from any neighboring cluster, it will be alone until it receives a “CM request” message from the same type of node. Steps explained are shown in Figure 11. The whole procedure explained for a CH and a CM is shown in the flowchart of the Figure 12.

**Figure 13.**
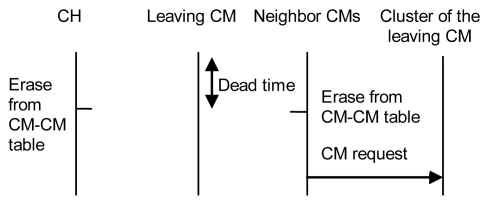
Protocol operation when a CM leaves the network.

When a CM leaves the cluster (because of its mobility, or due to failure or other issues), the CH of its cluster and its neighbor CMs will not receive any “keepalive” message from it. After a dead time, the CH will erase this entry from its CM table and its neighbor CMs from other clusters will erase this entry from their CM-CM table. If the CM from the other cluster does not have any other neighbor for this cluster, it will send a “CM request” message to that cluster. Figure 13 shows all steps explained.

**Figure 14.**
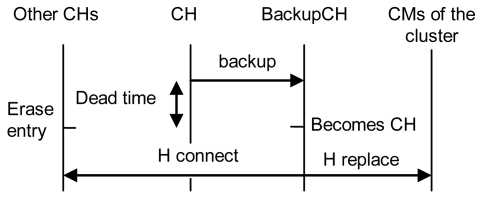
Protocol operation when a CH leaves the network.

Because the CH receives the *δ* parameter from all CMs in its cluster, it knows the best CM to promote in case of a failure or disconnection. The CM with highest δ parameter between the nearest ones is called “backup CH”. The CH sends the CM table and the CH table though “backup” messages to the backup CH. The first message has all the tables; the next ones will only be updates. The CH sends “keepalive H” messages periodically to its neighbor CHs and to the backup CH. If the CH fails down, the neighboring CHs and the backup CH will know it due to the absence of keepalive messages. If the backup CH does not receive a keepalive message from the CH for a dead time, it will become the CH its cluster. The neighbor CHs of the failed CH will erase that entry from their CH table. That update will be propagated through the CH network using the OLSR routing protocol (although it can be changed by other routing protocol). Because the backup CH has both CM and CH tables of the failed CH, it will become CH and will send a “H replace” message to all CMs and CHs in the table to indicate they have to replace the failed CH by the new one, so it establishes, for its first time, a connection with the CMs in its cluster. Figure 14 shows the described procedure.

### Protocol Messages

**Figure 15.**
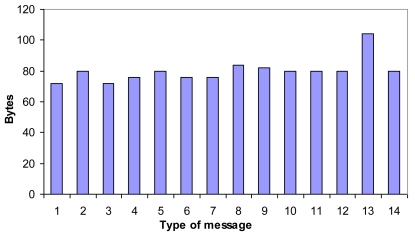
Bandwidth cost of the messages (backup CH message has two entries).

In order to achieve the proper operation of the architecture, 14 messages have been designed and developed. We have used 4 Bytes for *clusterID, nodeID*, l and d parameters and the node position, and 2 bytes for the type of message and the CM type. All are fixed size messages except the backup message that depends on the number of neighbors (although it is sent using incremental updates, there could be several new neighbors) and it is only sent when changes take place. Bandwidth cost in bytes for each message is shown in Figure 15 (1 = “Discovery”, 2 = “Discovery ACK”, 3 = “H connect”, 4 = “Welcome H”, 5 = “Keepalive H”, 6 = “M connect”, 7 = “Welcome M”, 8 = “Keepalive M”, 9 = “CM request”, 10 = “CM connect”, 11 = “Welcome CM”, 12 = “Keepalive MM”, 13 = “backup CH”, 14 = “H replace”). The messages' size is based on MAC layer in 802.11 and TCP/IP headers. The sum of these headers is 70 bytes.

## 6. Architecture Measurements

### 6.1. Test Bench

In order to measure our proposal, an application software has been developed, using Java programming, to run and test the designed protocol and the architecture performance. We programmed CH and CM functionalities. The application allows configuring some parameters such as the maximum number of connections, maximum distance, type of node, node position, keepalive time and so on). The application calculates λ and δ parameters internally. The MAC protocol used to measure the control messages was CSMA/CA in the frequency of 2.4 GHz. There was one laptop with a high gain antenna configured in a monitor mode that captured all data of the test bench using a sniffer application.

The test bench is formed by 16 Multisensors [33] in an open air environment. Because we needed a multisensory with high computation capacities, we used the Linksys WRT54GL router, from Cisco Systems inc., as the core controller. It is an embedded system that that allows us to connect several physical sensors in its serial interfaces. The Linksys WRT54G version 4.0 has a 200 Mhz processor, 4 Mbytes of flash memory, 16 Mbytes of RAM at 100 MHz clock rate and 256 Bytes prefetch cache. The Wireless interface accomplishes IEEE 802.11g at 54 Mbps and IEEE 802.11b at 11 Mbps standards. Its transmitting power is 18 dBm. It allows us to cover large distances.

The position of the multisensor is shown in Table 2a. X and Y values are in meters. It is one of the most representative test benches performed because it allows showing several operation procedures as they will be seen later. The maximum number of connections has been fixed to a value of four. The maximum distance to establish a connection with a CM of the same type was 100 meters. The keepalive time chosen was 30 seconds (it avoids too much energy consumption because of the number of messages sent and, if larger times are conFigured, nodes will discover the failures too late) and the dead time chosen was 60 seconds (twice the keepalive time). Sixty seconds seems to be enough time to know the node has failed. There are four types of nodes for each group. In order to make the test bench more understandable, CH nodes were CMs of the same type, but it does not affect to the system because they have to establish connections as CH and as CMs (the last ones are only established if the distance is lower than 100 meters, so in this case there will be none for type a). Then, the nodes are started in a sequential order every 10 seconds. This order is shown in Table 2a. Table 2b shows the connections that have been obtained for every node when the network has converged.

**Figure 16.**
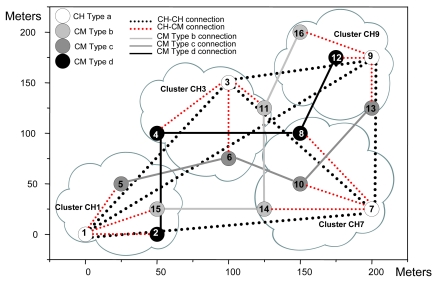
Nodes distribution and the connections established.

The topology obtained for the test bench and the physical position of the surrogates are shown in Figure 16.

CHs have connections with other CHs (lines formed by black points). CMs have a connection with the CH of its cluster (lines formed by red points) and with the selected CMs of the other clusters (solid lines in different tonalities of grey and in black). We have implemented the positions using only two dimensions, but our application supports three dimensions.

### 6.2. Network Measurements

In order to check the performance of the developed architecture, the behavior of the nodes in the initialization phase has been measured for 10 minutes. It allows us to see how the network performs when the nodes join in. It gives us the amount of control traffic introduced by our architecture. The following graphs are the most representative measurements obtained over multiple experiments. We have performed several times the same topology obtaining very close results (we think that their difference is given because of the operative system response or due to electronic issues).

**Figure 17.**
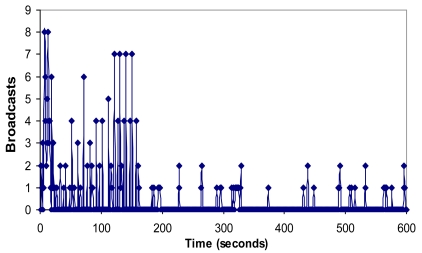
Number of broadcasts per second.

It can be observed that when all these nodes started sequentially, only 4 groups were created and the CHs were nodes 1, 3, 7 and 9. The number of broadcasts sent by the nodes when the network is setting up is shown in Figure 17. There are broadcast peaks due to new joining nodes in the first 160 seconds, but the highest peaks had eight broadcasts. There are no more than two broadcasts per second when the network is stabilized. So it demonstrates that there is low bandwidth consumption and little energy is wasted because there are few broadcasts.

**Figure 18.**
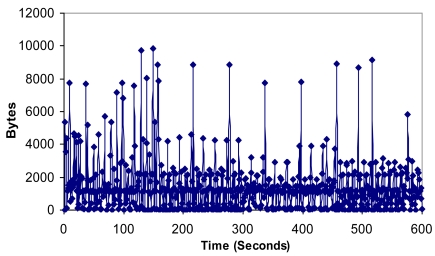
Number of bytes per second.

Figure 18 shows the number of bytes/s in the network. At the beginning there are many peaks because of keepalive messages and joining nodes in the initial process. When the network has converged, there are peaks approximately every 60 seconds because of keepalive messages, but the number of bytes/s is very similar. It demonstrates that little additional traffic will be introduced when the topology changes. On the other hand, there is a mean value of 1,482.5 Bytes per second, with a maximum value of 9,823 bytes per second and a minimum value of zero.

Taking into account that the test bench was performed in an IEEE 802.11g Wireless LAN with 54 Mbps (16 nodes in 200 × 200 meters), we can state that, when our protocol is running, the limitation of number of devices in the network will be given by the overheads and timing constraints from all other network layers, not by our protocol.

**Figure 19.**
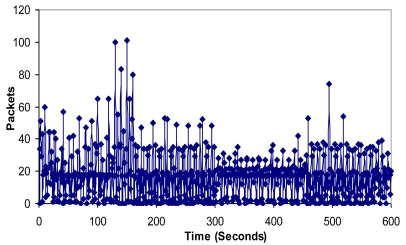
Number of packets per second.

It can be seen that there are more packets per second in the architecture when there are more clusters in the network (between 140 and 160 seconds can be seen in Figure 19). It is because new CMs request neighbor CMs of other clusters. Once the network has converged, there are not so much variations. The mean value has been 17.41 packets per second. We obtained a maximum value of 101 packets per second.

### 6.3. Fault Tolerant Procedure

This subsection shows the procedure and the connections obtained after a node failure. It is divided into three scenarios to show different cases. The test bench shown in Figure 16 has been used as a starting point. The multisensors have had the same initial parameters as in Subsection 5.1. All multisensors have had the same starting energy for simplicity and they have appeared in the scenario in the same manner as in Subsection 6.1. The traffic obtained in the whole process is not too relevant because there are very few messages transmitted through the network and they cannot be distinguished between the traffic measured.

In the first scenario node 3 fails down one second after it sends its update message. Then, we sniffed the open air during 140 seconds (60 seconds was the conFigured dead time interval). This time makes us certain that node 4 had noticed that node 3 had failed. It also gives us enough time to compare the update messages with the ones sent 120 seconds later.

**Figure 20.**
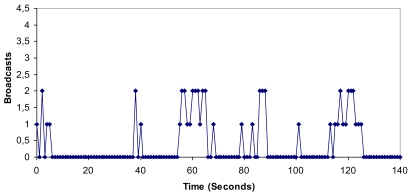
Number of broadcasts per second when node 3 fails.

**Figure 21.**
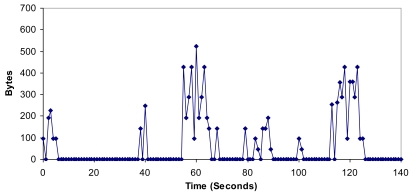
Number of bytes per second when node 3 fails.

**Figure 22.**
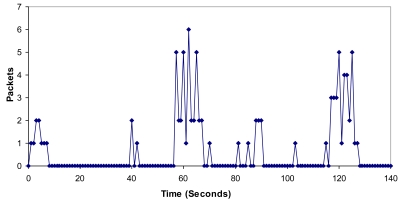
Number of packets per second when node 3 fails.

Figures 20–22 show the measurements gathered. We observe that there are more broadcasts/s, bytes/s and packets/s sent to the network between seconds 55 and 65 than between 115 and 125. We can see in Figure 20 that there were 18 broadcasts/s in the first range *versus* 15 broadcasts/s in the second range.

**Figure 23.**
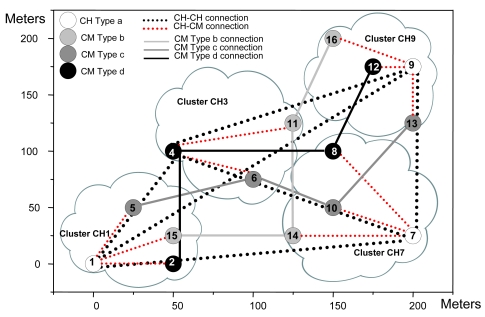
New topology when node 3 fails.

Figure 21 shows that the first range has 3,196 bytes/s sent to the network, while the second range has 3,054 bytes/s. In Figure 22, 34 packets were sent in the first range while 32 packets were sent in the second range. The measurements taken let us know that, although there were more broadcasts/s, bytes/s and packets/s, the rise in the first range has been moderated, so when a node fails down, there is very low impact in the network. It agrees the protocol operation shown in Figure 14. As all nodes have had the same initial energy, taking into account Equation 1, it was deduced that node 4 would be the new CH because it had lower *nodeID*. Figure 23 shows the topology when the network has converged. Because node 3 only had connections as a CH, but not as a CM, when the network has converged, there is just one connection less, so it keeps the same stability. The steps followed to achieve the new topology are shown in Figure 14.

In the second scenario node 15 fails one second after it sends its update message. Then, we sniffed the open air during 140 seconds (60 seconds was the conFigured dead time interval). This time makes us certain that node 14 had noticed that node 15 had failed. It also gives us enough time to compare the update messages with the ones sent 120 seconds later.

**Figure 24.**
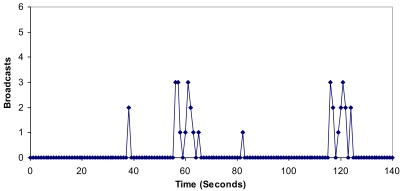
Number of Broadcast per second when node 15 fails.

**Figure 25.**
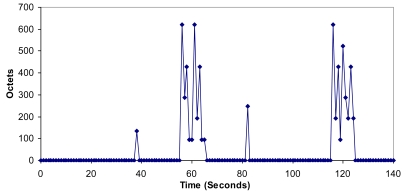
Number of Bytes per second when node 15 fails.

**Figure 26.**
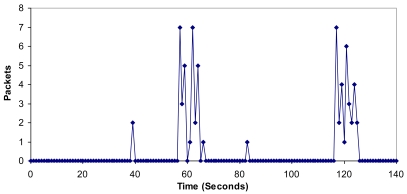
Number of Packets per second when node 15 fails.

Figures 24–26 show the measurements obtained when node 15 fails. There is the same number of broadcasts/s, bytes/s and packets/s sent to the network between seconds 55 and 65 than between 115 and 125. In both ranges, we measured 15 broadcasts/s, 2,958 bytes/s and 30 packets/s respectively. The measurements taken show that a CM failure does not have any impact in the network.

**Figure 27.**
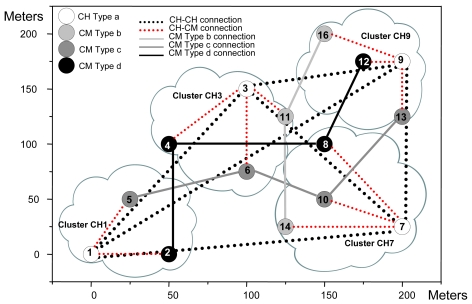
New topology when node 15 fails.

Figure 27 shows the network when it has converged. Now, we observed that there were two connections less: the connection between node 14 and node 15 and the connection between node 1 and node 15. It can be seen that there was no other type b node in the cluster CH1 to replace it and there are no other type b nodes close to it to have connections.

**Figure 28.**
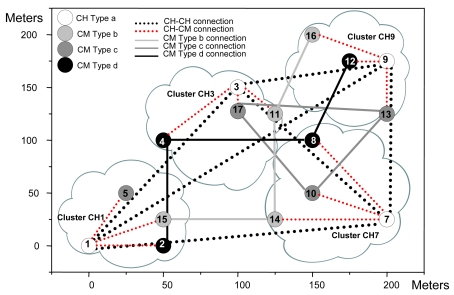
New topology when node 6 fails.

In the third scenario node 6 fails. After a second, we introduced node 17 in position (100, 125), which is a CM of type c in order to provide a backup for cluster CH3. When we saw the connections in the network (see Figure 28), we discovered that the new node has nodes 10 and 13 with distances lower than 100 meters, but node 5 was at a longer distance (it was not within the coverage area). In this case we don't provide broadcasts/s, bytes/s and packets/s measurements because they were similar to the one obtained when node 15 fails.

### 6.4. Bandwidth, Jitter, Delay, Lost Packets and Number of Packets with Errors

**Figure 29.**
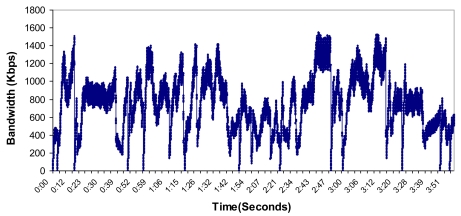
Bandwidth consumed during the test.

We took as a starting point the topology shown in Figure 16, but this time it was performed in a hard indoor environment with many WiFi networks working in parallel. In order to take these measurements, a Wireshark network protocol analyzer was used [34]. First, we sent variable bitrate streams during four minutes from node 5 to node 13. The path followed was: node 5 – node 6 – node 10 – node 13. The bandwidth consumed is shown in Figure 29. It varied from 0 to 1551.26 Kbps. The mean value was 750.81 Kbps.

**Figure 30.**
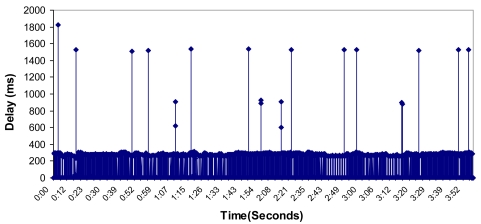
Delay measured during the test.

The measured delay is shown in Figure 30. We obtained a mean value of 19.16 milliseconds (which is a real time values because it is lower than 50 milliseconds). The delay varied from 0 to 1,826.41 milliseconds.

**Figure 31.**
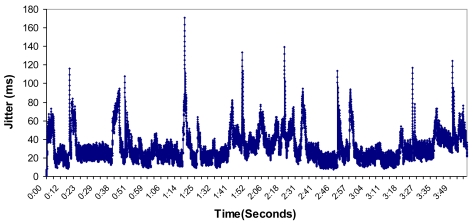
Jitter measured during the test.

Then, we measured the jitter of the packets during the test. The result is shown in Figure 31. We obtained a mean value of 31.11 milliseconds. The maximum value was 171.14 milliseconds and the minimum value was 0 milliseconds.

During this test, 15,537 packets were sent, 1,475 of them were lost (9.49% of dropped packets), and 183 of them had sequence errors. Those values are not so bad if we take into account that eight IEEE 802.11b/g networks were detected in the place where we performed the test. Packet loss can be caused by any of the following: signal degradation over the air, oversaturated network links, corrupted packets rejected in-transit, faulty networking hardware, faulty network drivers or normal routing routines.

## 7. Protocol Comparisons

Cluster architectures have been proposed for many different purposes. All of them have the following benefits in common:

- Topology updates overhead reduction.
- The clustering structure is self-organized and adaptable.
- Fully distributed operation.
- New nodes do not have to be searched or initiated.
- Broken routes cloud is repaired locally without rediscovery.
- Reduction in the energy dissipation.
- Broadcasts are done only by the boundary nodes.
- The routing is source initiated.
- Lower memory overhead.
- Quite scalable.

There are many other characteristics that could be different. The cluster scheme can be applied in many different manners in order to achieve diverse benefits. This section compares our proposal with others in existence in order to show the benefits of our protocol. Only those architectures whose protocol has been available and accessible are included.

### 7.1. Architecture Comparison

Krishna *et al.* presented in [35] a methodology for routing and topology information maintenance in mobile wireless network based on the existence of clusters in random graphs. They divided the graph into a number of overlapping clusters. There are no cluster heads in the proposal. It is an on-demand source routing. The performance of the routing protocol proposed by them is determined by the average cluster size. The effectiveness of their approach lies in the fact that any routing protocol can be directly applied to the network, replacing the nodes by clusters. They designed nine messages, but their approach has to be implemented over another routing protocol. They proposed a standard distance vector routing protocol to apply their proposal. Taking into account that AODV has seven messages. Their implementation needs 16 messages.

CBRP (cluster-based Routing protocol) was proposed in [6]. The protocol divides the nodes of the *ad hoc* network into a number of overlapping or disjoint 2-hop-diameter clusters in a distributed manner. CBRP uses IP Protocol for routing purposes and interoperability with fixed networks. It uses six messages plus the ARP messages, so it needs eight messages to work properly. As a source routing protocol, there is an overhead of bytes per packet.

In [36], the authors proposed a Cluster-Based Security Architecture for *ad hoc* networks. They proposed a division of the network into clusters, with one special head node each, for a distributed public key infrastructure. These cluster head nodes execute administrative functions and hold shares of a network key used for certification.

KCLS protocol was proposed in [37]. The paper describes a location-service protocol based on the clustering architecture, which is able to balance the tradeoff between the communication overheads and the accuracy of location information. It has the capability of cluster-level self-route recovery against interlink failures. Taking into account that KCLS is based on the KCMBC protocol and on a Link State protocol, it needs 13 messages plus the protocol needed to acquire their position using GPS.

In [9] the authors proposed an adaptive clustering scheme for spatial reuse of the bandwidth (relying on a code division access scheme) for multimedia support in Mobile Wireless Networks. They use only one code within each cluster. The clusters are independently controlled and are dynamically reconFigured as nodes move. Bandwidth can be shared or reserved in a controlled fashion in each cluster.

LEACH (Low-Energy Adaptive Clustering Hierarchy) protocol was proposed in [34]. It is a clustering-based protocol that utilizes randomized rotation of local cluster-heads minimizing the global energy usage by evenly distributing the energy load among the sensors in the network. LEACH uses localized coordination and incorporates data fusion into the routing protocol to reduce the amount of information that must be transmitted to the base station. It is completely distributed, requiring no control information from the base station, and the nodes do not require knowledge of the global network in order to operate.

Reference [38] presented the “Base Station Controlled Dynamic Clustering Protocol” (BCDCP). Their proposal utilizes a high-energy base station to set up clusters and routing paths, perform randomized rotation of cluster heads to avoid cluster head overload, and carry out other energy-intensive tasks. It distributes the energy dissipation evenly among all sensor nodes to improve network lifetime and average energy savings.

In [39], authors proposed CLACR (Core Location-Aided Cluster-Based Routing Protocol for Mobile *Ad Hoc* Networks). CLACR splits the network into square clusters. Cluster heads compute the desired route using Dijkstra algorithm, which reduces the number of nodes participating in routing, the routing traffic and the route setup time.

CBLARHM (Cluster Based Location-Aware Routing Protocol for Large Scale Heterogeneous MANET) was proposed in [40]. The system uses the geographical location information of mobile nodes, provided by global positioning systems (GPS), to confine the route searching space, for the specified destination node.

WCA (A Weighted Clustering Algorithm for Mobile *Ad Hoc* Networks) was presented in [41]. They proposed an on-demand, weight-based distributed clustering algorithm that takes into consideration the ideal degree, transmission power, mobility, and battery power of mobile nodes. The clustering algorithm tries to distribute the load as much as possible aimed to reduce the computation and communication costs. The algorithm is executed only when there is a demand, *i.e.*, when a node is no longer able to attach itself to any of the existing cluster-heads.

In [42] CLTC was presented, a Cluster-Based Topology Control Framework for *Ad Hoc* Networks. CLTC uses a centralized algorithm within a cluster and between adjacent clusters to achieve strong connectivity. It utilizes a hybrid approach to control the topology using transmission power adjustment and yet achieves the scalability and adaptability of a distributed approach with localized information exchange between adjacent clusters. CLTC framework guarantees global k-connectivity as long as the original topology is k-connected.

Table 3 compares the described protocols. The number of messages of some protocols is provided by the explanations of their authors in the referenced paper. Some of them do not take into account messages such as the new nodes messages or messages to provide fault tolerance, or are based on other algorithms not described in the paper (although in some cases we have found these and taken them into account). Many other proposals have been found in the literature, but they have not been included because of the lack of sufficient information in the original publication to fill in the rows of Table 3, or because the authors just described the algorithm, not the protocol, or because they are slight extensions of the proposals shown in the Table. Several features in our proposal may be highlighted. First, it is the only one that is able to use several routing protocols in the same network, and it does not depend on a specific routing protocol so it could be adapted to the environment issues. It does not have too many messages compared with the other ones (despite the simplicity of the CBRP, some procedures are not explained and it does not provide fault tolerance). Second, it is the only one where a new node selects the cluster not only by the proximity or radio signal strength but also takes into account the available capacity of the neighbors (which depends on the available energy). Third, it is the only one that has been proposed to create parallel networks. Our proposal has been designed to provide fault tolerance and our design is described in detail giving all the messages needed to run properly.

There are several differences in the metric used to elect the Cluster Head. Some of them use the lowest *NodeID* or their position, while others use just a random system. Others are not explained or propose a weak system. Between the most complex metrics, we distinguish CBLARHM that uses a node-weight heuristic parameter, based on the ideal number of nodes in a cluster, the battery power, the average link stability and the average dependency probability, to elect the head cluster node, but this is very impractical because it is difficult to determine the value of some of these parameters.

WCA uses a combined weight metric based on the ideal node degree, transmission power, mobility and the battery power of the nodes. It is a good idea and seems very useful, but they propose this metric for a cluster-based general purpose algorithm, and maybe these parameters are good for a specific case, but not for all cases, as some parameters could be missing such as the node's position or the node's load. Our metric does not take mobility into account since the entire cluster could be moving and avoids continually selecting the motionless nodes. On the other hand, it does take into account the more stable node in the cluster and its energy, thus making the system very simple and practical.

### 7.2. Measurement Comparisons

First of all, we want to emphasize that all works found in the literature provide only measurements taken from simulations, not from real deployments nor from controlled testbeds. Table 4 gives the type of measurements provided by several papers in the literature. Some of them are focused on measuring parameters related with the cluster size and the number of clusters. Due to cluster-based networks are mostly used for energy saving, most of them simulate energy issues.

Taking into account that the more message transmissions, the more energy dissipation, only CBRP [6] and CLACR [38] (and may be CLTC [41], depending on the number of messages transmitted using GPS) will consume less energy than our proposal.

Taking into account the measurements provided in this paper, we will compare our measurements with some measurements provided in other authors in their works. We are not going to implement the same test bench, we will just take their measurements and compare them with our measurements in some particular cases.

Studying the results presented by Bechler *et al.* in [35], taking into account the same keepalive interval (30 seconds), we obtained an average value of 17.41 packets per second for a topology of three hops, while they obtained higher values (between 50 and 100 packets per second) in a random topology with 15 nodes. In terms of overhead (packets per second), it gives us an improvement of more than 65.2 % compared the best case of their protocol.

Hollerung presented in [45] several graphs that show the packet delivery ratio versus the number of nodes in the cluster network. Close to 1 packet delivery ratio (0.99 packet delivery ratio) was shown for 25 nodes, while we can see that it agrees our measurements. Once our network has converged (after the setup phase), we obtain 15.9 packets/s for 16 nodes. It gives us almost the same packet delivery ration. The worst case has been presented in [41] by Shen *et al.* because they measured 2.5 messages per node for 100 nodes.

In Subsection 6.4 we have obtained a mean value of 19.16 milliseconds when there were three hops between the source and the destination. In [30] we can see that the average delay for three hops was higher than 500 milliseconds in the best case (and lower than 1,500 milliseconds in the worst case).

## 8. Conclusions

The paper shows the development of an architecture that creates clusters and establishes connections between sensor of the same type by building different sensor networks. Cluster heads manage the network since they have connections with other cluster heads and these connections allow connecting cluster members from different clusters. Cluster members of the same type form a specialized network. Although there are several proposals of cluster-based systems in existence, the novelty of our proposal is that it could be used to build different networks with different routing protocols, while other cluster-based networks can run just one routing protocol and can build only one type of network. One of the main goals is that if all cluster heads switch off at the same time, the system is able to continue working, although there will not be new connections between clusters through CHs.

In this paper, the description of the protocol developed and the flow of the messages have been presented. The performance of the network and how nodes perform in different execution cases have been shown. It has been demonstrated that the architecture requires low bandwidth to run and work properly. We are currently implementing the protocol in an embedded sensor.

Comparing our proposal with others, it can be seen that the detailed description of our protocol allows its easy implementation. Other protocols compared in this paper do not take into account the discovery algorithm, and/or the node starting procedure, and/or the fault tolerance and/or even some parts of their proposal are not described in detail, so more messages that are not included in their description will be needed.

This paper also starts a new research line because, in many cases, not all sensors can interact with the others in the same networks, so, in these cases, different sensor networks are needed and there is no other architecture in existence to support it.

One of the main contributions is that the proposed system allows the creation of virtual wireless sensor networks and the wireless sensor networks virtualization. As far as we know, it has not been introduced before in the scientific field.

## Figures and Tables

**Table 1.** Diameter for each type of cluster (*k* is the number of clusters and *P* is the number of levels of the hierarchy).

**Type of cluster**	**Diameter (*d*)**
1-level cluster	*K + 1*
*P*-level cluster	2·*p*
Planar cluster with one hop	3·*k* – 1
Our proposal	*K* – 1

**Table 2.** Nodes' positions.

**Node number**	**Type of node**	**X**	**Y**
1	a	0	0
2	b	50	0
3	a	100	150
4	b	50	100
5	c	25	50
6	c	100	75
7	a	200	25
8	b	150	100
9	a	200	175
10	c	150	50
11	d	125	125
12	b	175	175
13	c	200	75
14	d	125	25
15	d	50	25

**Table 2b**. Neighbor connections.			

**Node number**	**Role**	**Connections with CH**	**Connections with CM**
1	CH	3, 7, 9	2, 5, 15
2	CM	1	4
3	CH	1, 7, 9	4, 6, 11
4	CM	3	2, 8
5	CM	1	6
6	CM	3	5, 10
7	CH	1, 3, 9	8, 10, 14
8	CM	7	4, 12
9	CH	1, 3, 7	12, 13, 16
10	CM	7	6, 13
11	CM	3	14, 16
12	CM	9	8
13	CM	9	10
14	CM	7	11, 15
15	CM	1	14
16	CM	9	11

**Table 3.** Cluster architectures comparison.

**Architecture**	**Overlapping nodes**	**Uses other routing protocols**	**Number of messages**	**New node cluster's selection**	**Purpose**	**Node Fault Tolerance**	**Cluster Head selection**
P. Krishna *et al.* [35]	Yes	Just one at a time	16	Proximity	Routing	No	n/a
CBRP [6]	Yes	No	6	nodeID	Routing	No	Lowest node ID
Marc Bechler *et al.* [36]	No	Just one at a time	n/p	n/p	Security	Yes, but very weak	Trusted node, but not explained what happens if there are several trusted nodes.
KCLS [37]	No	No	13 + GPS protocol	Distance to the head node less than k hops	Location Service	Yes	Mobility threshold
Chunhung R. Lin [9]	No	Just one at a time	n/p	nodeID	Bandwidth allocation	Yes	Lowest node ID
LEACH [34]	No	No	n/p	received signal strength	Energy optimization	No	Random rotation
BCDCP [38]	No	No	n/p	Location	Energy optimization	No	Random
CLACR [39]	No	No	11	Location	Routing	Just for location servers	Closest to the cluster center position
CBLARHM [40]	No	No	17+ GPS	Relative distance, velocity and time	Routing	No	Node-Weight heuristic
WCA [41]	No	Just one at a time	n/p	Degree difference and proximity	General purpose	No	Combined weight metric
CLTC [42]	No	Just one at a time	10+ GPS	Coverage area	Topology control	No	n/p
Our proposal	No	Yes and it could use many simultaneously.	14	Proximity + capacity parameter	Create parallel networks	Yes	Promotion Parameter

Note: n/a means not applicable, n/p means not provided.

**Table 4.** Type of measurements provided by other authors.

**Reference**	**Type of measurements**	**Purpose**
[34]	It gives the simulations of the average cluster size and the number of clusters versus the degree of the nodes.	Parameters related with the cluster size and the number of clusters
[40]	It gives the average number of cluster heads versus the number of nodes and the cluster size versus the number of nodes.	
[42]	It gives the average number of clusters versus the transmission range or the maximum displacements	
[13]	It provides the energy consumption versus the distance to the nodes and the number of nodes, the number of alive nodes versus the round and the energy consumed versus the number of rounds.	Energy issues
[19]	It shows the energy consumption versus the number of L-sensors, average number of working nodes versus the number of sensors and the energy consumption versus the average desired coverage degree.	
[32]	It gives the number of nodes alive versus the time, the energy dissipation versus the percentage of nodes that are cluster heads, and the energy dissipation versus the network diameter.	
[37]	It compares several cluster-based protocols versus the number of rounds, number of messages received versus the energy dissipation and energy consumed and the number of nodes alive as a function of network area.	
[43]	It shows the measurements of the number of alive nodes versus the time, the energy dissipation versus the time and the energy dissipation in the setup phase.	
[44]	It shows the percentage of energy consumed versus the cluster radius, the average cluster head residual energy versus the cluster radius and the cluster energy dissipated versus the number of nodes.	

## References

[1] Yick J., Mukherjee B., Dipak D. (2008). Wireless Sensor Network Survey. Comput. Netw..

[2] Krco S. (2005). Health Care Sensor Networks—Architecture and Protocols. Ad Hoc. Sensor Wireless Networks.

[3] Mainwaring A., Szewczyk R., Anderson J., Polastre J. Habitat Monitoring on Great Duck Island.

[4] Summers S.A. (2006). Wireless Sensor Networks for Firefighting and Fire Investigation.

[5] Yang H., Sikdar B. A Protocol for Tracking Mobile Targets Using Sensor Networks.

[6] Jiang M., Li J., Tay Y.C., Cluster Based Routing Protocol (CBRP) (1998). http://tools.ietf.org/html/draft-ietf-manet-cbrp-spec-01.txt.

[7] Ghosh R.K., Garg V., Meitei M., Raman S., Kumar A., Tewari N. (2006). Dense Cluster Gateway Based Routing Protocol for Multi-Hop Mobile *Ad Hoc* Networks. Ad Hoc Netw..

[8] Yu J.Y., Chong P.H.J. (2005). A Survey of Clustering Schemes for Mobile *Ad Hoc* Networks. IEEE Commun. Surv. Tutorials.

[9] Lin C.R., Gerla M. (1997). Adaptive Clustering for Mobile Wireless Networks. IEEE J. Sel. Areas Commun..

[10] Ryu J.H., Song S., Cho D.H. New Clustering Schemes for Energy Conservation in Two-Tiered Mobile *Ad Hoc* Networks.

[11] Chatterjee M., Das S.K., Turgut D. An On-Demand Weighted Clustering Algorithm (WCA) for *Ad Hoc* Networks.

[12] Ohta T., Inoue S., Kakuda Y. An Adaptive Multihop Clustering Scheme for Highly Mobile *Ad Hoc* Networks.

[13] Jin Y., Wang L., Kim Y., Yang X. (2008). EEMC: An Energy-efficient Multi-level Clustering Algorithm For Large-scale Wireless Sensor Networks. Comput. Netw..

[14] Das B., Bharghavan V. Routing in *Ad Hoc* Networks Using Minimum Connected Dominating Sets.

[15] Basu P., Khan N., Little T.D.C. A Mobility Based Metric for Clustering in Mobile *Ad Hoc* Networks.

[16] Ren Q.C., Liang Q.L. (2006). Energy-Efficient Medium Access Control Protocols for Wireless Sensor Networks. EURASIP J. Wirel. Commun. Netw..

[17] Amis A.D., Prakash R. Load-Balancing Clusters in Wireless *Ad Hoc* Networks.

[18] Abbasi A.A., Younis M. (2007). A Survey on Clustering Algorithms for Wireless Sensor Networks. Comput. Netw..

[19] Du X., Lin F. (2005). Maintaining Differentiated Coverage in Heterogeneous Sensor Networks. EURASIP J. Wirel. Commun. Netw..

[20] Zou Y., Chakrabarty K. (2004). Sensor Deployment and Target Localization in Distributed Sensor Networks. ACM Trans. Embed. Comput. Syst..

[21] Yuan Y., Chen M., Kwon T. (2006). A Novel Cluster-Based Cooperative MIMO Scheme for Multi-Hop Wireless Sensor Networks. EURASIP J. Wirel. Commun. Netw..

[22] Ibriq J., Mahgoud I. Cluster-Based Routing in Wireless Sensor Networks: Issues and Challenges.

[23] Lee K.H., Han S.B., Suh H.S., Lee S.K., Hwang C.S. (2005). Authentication Based on Multilayer Clustering in *Ad Hoc* Networks. EURASIP J. Wirel. Commun. Netw..

[24] Kredo K., Mohapatra P. (2007). Medium Access Control in Wireless Sensor Networks. Comput. Netw..

[25] Lloret J., Boronat F., Palau C., Esteve M. Two Levels SPF-Based System to Interconnect Partially Decentralized P2P File Sharing Networks.

[26] Clausen T., Jacquet P. (2003). Optimized Link State Routing Protocol (OLSR). RFC 3626.

[27] Perkins C., Belding-Royer E., Das S. (2003). *Ad Hoc* On-Demand Distance Vector (AODV) Routing. RFC 3561.

[28] Johnson D., Hu Y., Maltz D. (2007). The Dynamic Source Routing Protocol (DSR) for Mobile *Ad hoc* Networks for IPv4. RFC 4728.

[29] Park V., Corson S. (2001). Temporally-Ordered Routing Algorithm (TORA) Version 1, Functional Specification, Internet Draft. http://tools.ietf.org/id/draft-ietf-manet-tora-spec-04.txt.

[30] Chang Y.C., Lin Z.S., Chen J.L. (2006). Cluster Based Self-Organization Management Protocols for Wireless Sensor Networks. IEEE Trans. Consum. Electron..

[31] Lloret J., Tomas J., Garcia M., Canovas A. (2009). A Hybrid Stochastic Approach for Self-Location of Wireless Sensors in Indoor Environments. Sensors.

[32] Heinzelman W.R., Chandrakasan A., Balakrishnan H. Energy-efficient communication Protocol for Wireless Microsensor Networks.

[33] Bri D., Coll H., Garcia M., Lloret J. A Multisensor Proposal for Wireless Sensor Networks.

[34] Wireshark Network Protocol Analyzer website http://www.wireshark.org.

[35] Krishna P., Vaidya N.H., Chatterjee M., Pradhan D.K. (1997). A Cluster-Based Approach for Routing in Dynamic Networks. ACM SIGCOMM Comput. Commun. Rev..

[36] Bechler M., Hof H., Kraft D., Pählke F., Wolf L. A Cluster-Based Security Architecture for *Ad Hoc* Networks.

[37] Leng S., Zhang L., Fu H., Yang J. (2007). A Novel Location-Service Protocol Based on k-Hop Clustering for Mobile *Ad Hoc* Networks. IEEE Trans. Veh. Technol..

[38] Muruganathan S.D., Ma D.C.F., Bhasin R.I., Fapojuwo A.O. (2005). A Centralized Energy-Efficient Routing Protocol for Wireless Sensor Networks. IEEE Commun. Mag..

[39] Shih T.F., Yen H.C. Core Location-Aided Cluster-Based Routing Protocol for Mobile *Ad Hoc* Networks.

[40] Wang Y., Chen H., Yang X., Zhang D. Cluster Based Location-Aware Routing Protocol for Large Scale Heterogeneous MANET.

[41] Chatterjee M., Das S.K., Turgut D. (2002). WCA: A Weighted Clustering Algorithm for Mobile *Ad Hoc* Networks. Cluster Comput..

[42] Shen C., Srisathapornphat C., Liu R., Huang Z., Jaikaeo C., Lloyd E. (2004). CLTC: A Cluster-based Topology Control Framework for *Ad Hoc* Networks. IEEE Trans. Mob. Comput..

[43] Wen C.Y., Sethares W.A. (2005). Automatic Decentralized Clustering for Wireless Sensor Networks. EURASIP J. Wirel. Commun. Netw..

[44] Chen J., Yu F. A Uniformly Distributed Adaptive Clustering Hierarchy Routing Protocol.

[45] Younis O., Fahmy S. Distributed Clustering in *Ad Hoc* Sensor Networks: A Hybrid, Energy-Efficient Approach.

[46] Hollerung T.D. (2004). The Cluster-Based Routing Protocol.

